# An Innovative Approach to Accelerate Maxillary Canine Retraction With Low-Amperage Direct Electric Current: A Preliminary Clinical Study

**DOI:** 10.7759/cureus.80573

**Published:** 2025-03-14

**Authors:** Mohammad N. Kheshfeh, Mohammad Y. Hajeer, Ahmad S. Burhan, Mowaffak A. Ajaj, Samer T. Jaber, Mhd Firas Al Hinnawi

**Affiliations:** 1 Department of Orthodontics, Faculty of Dentistry, University of Damascus, Damascus, SYR; 2 Department of Orthodontics, Faculty of Dentistry, Al-Watanyia Private University, Hama, SYR; 3 Department of Biomedical Engineering, Faculty of Electrical and Mechanical Engineering, University of Damascus, Damascus, SYR

**Keywords:** acceleration of orthodontic tooth movement, acceptability, anterior teeth retraction, canine retraction, electrical stimulation, low-amperage direct electric current, low-intensity electric stimulation, orthodontic tooth movement rate, patient-reported outcomes, two-step retraction

## Abstract

Background and objective

Accelerating orthodontic tooth movement is a significant goal for clinicians and patients. This study aimed to investigate the effectiveness of a low-ampere, electrically inducible tooth movement accelerator device. The study focuses on an innovative intraoral removable device designed to expedite the retraction of maxillary canines.

Methods

This research was conducted in the Orthodontics Department at Damascus University, Syria, between September 2022 and March 2023, and involved seven patients (five females and two males; mean age: 20.85 ± 1.34 years) initially diagnosed with class II, division I malocclusion. The treatment plan included retraction of the upper anterior teeth in two stages following the extraction of the first premolars on each side. Electrical stimulation was applied during the retraction of the maxillary canines using a removable, custom-manufactured device developed by the researchers. Patients were instructed to wear the device for five hours daily. The variables investigated included the rate of canine retraction, safety, and patient acceptance of the device.

Results

The average rate of canine retraction was consistent, averaging 1.25 ± 0.13 mm/month. No side effects, such as irritation or discomfort, were observed. Patients reported no adverse effects and stated that they would recommend the technique to others. Five out of seven patients found it easy to adapt to the device, while two found it moderately challenging.

Conclusions

Our findings showed that low-amperage direct current electrical stimulation effectively accelerated orthodontic movement. The maxillary canine retraction was significantly sped up without any adverse effects. Patients responded positively, indicating feasibility and acceptability. This technique could reduce treatment duration, which is pending further validation.

## Introduction

During the treatment of class II cases in adults with the camouflage technique, the first premolars are the most common teeth to be extracted, followed by the retraction of the canines and then the incisors using the two-stage retraction technique [1]. This approach often extends the duration of orthodontic treatment to 24-36 months, presenting a significant challenge [2,3]. Given the numerous complications associated with prolonged orthodontic treatment, such as caries and periodontal infections, patients and orthodontists have strongly desired to expedite the process [4,5].

Researchers have explored various methods to accelerate orthodontic tooth movement, both non-surgical and surgical, with the surgical procedure being the most widely used and clinically tested [6]. Several surgical techniques, such as osteotomy [7], corticotomy [2], interseptal alveolar surgery, periodontal ligament distraction [8], osteoperforations [9], and piezocision [6], have been developed to reduce the total treatment time. The effectiveness of these surgical methods has been proven over the last two decades. Non-surgical methods have also been investigated given the undesirability of non-orthodontic surgical procedures in doctors and patients [10]. Physical non-surgical methods include the application of high-intensity laser to perforate the alveolar cortical bone, accelerating canine retraction by 32.55% [11], low-intensity laser [12], and the use of pulsed electromagnetic fields, which have shown success in accelerating orthodontic tooth movement [13]. Another promising method involves microcurrent electrical stimulation, a relatively new technique that has not yet gained widespread attention in dentistry [14,15].

Several animal studies have demonstrated that combining MET with mechanical force significantly speeds up tooth movement compared to orthodontic forces alone [16]. In a study by Kim et al. (2008), this technique was clinically applied to retract canines [17]. The study concluded that applying a small external electric current from a miniature device could accelerate orthodontic tooth movement by approximately 30%, potentially reducing the overall duration of orthodontic treatment. However, the study has been criticized for its small sample size (only seven patients), having a short follow-up period of just four weeks, and the fixed and portable design of the device on the canine bracket, which raises doubts about its stability, the patient's oral care, and the possibility of significant and continuous irritation of the inside of the cheek [17]. The main challenge preventing the widespread use of electrical stimulation to accelerate orthodontic tooth movement has been effectively applying it inside the mouth [18].

In light of this, the research team in the Department of Orthodontics, Damascus University, alongside an electronic engineer, designed an innovative intraoral electromotor device to address this issue. The project aimed to employ an easily applicable device that generates the suitable electrical current for the necessary duration with minimal complications, aiming to stimulate and accelerate orthodontic tooth movement during maxillary canine retraction. This preliminary report investigates the safety and efficacy of this novel electromotor device in accelerating maxillary canine retraction.

## Materials and methods

Study design and setting

This research entailed an initial clinical experimental study to evaluate the effectiveness of a new device designed to accelerate tooth movement in orthodontics. The study was conducted in the Orthodontics Department, Damascus University, Faculty of Dentistry, Syria, between September 2022 and March 2023. The study protocol received approval from the Biomedical Research Ethics Committee of Damascus University (DN-30082022-9) before the commencement of this trial. Our University regulations deemed that no registration with clinical trial registries was required given the nature of this study (i.e., a pilot study with no control group).

Patient recruitment

Seven patients diagnosed with class II, division I malocclusion were selected from those referred to the Orthodontics Department, Damascus University, Faculty of Dentistry, between June 2022 and August 2022. Inclusion criteria encompassed patients aged 18-28 years with class II, division I malocclusion, and a treatment plan involving the two-step retraction of the anterior teeth following the extraction of both upper first premolars. Exclusion criteria included (i) severe overjet (over 10 mm), (ii) extreme dental crowding, (iii) missing teeth in the maxilla, (iv) previous orthodontic intervention, (v) patients undergoing any general drug treatment, and (vi) gingival diseases, (vii) poor oral hygiene, and (viii) smoking, as tobacco smoke and high temperatures can increase mucosal keratinization, potentially increasing resistance to electronic current flow [19]. After the protocol was explained to the patients, none refused participation, and all provided written informed consent.

Treatment sequence

Anchorage Reinforcement

Anchorage reinforcement was achieved using a transpalatal arch on the first molars.

Leveling and Alignment

This study utilized the Pinnacle™ MBT prescription and pre-adjusted fixed orthodontic appliances from Ortho Technology (West Columbia, SC). The archwires, sourced from Ortho Technology, were replaced every 21 days with the following sequence: 0.012-inch NiTi, 0.014-inch NiTi, 0.016-inch NiTi, 0.016 × 0.012-inch NiTi, 0.017 × 0.025-inch NiTi, 0.019 × 0.025-inch NiTi, and finally, 0.019 × 0.025-inch stainless steel (SS).

Extraction of First Premolars

The maxillary first premolars were extracted before placing the 0.017 × 0.025 NITI Wire in the leveling and alignment phase of the treatment.

Canine Retraction and Application of Low-Amperage Direct Electric Current (DC)

A force of 150 g will be applied using a closed spring that extends from the upper canine bracket to the upper first molar hook via an intraoral spring (NT3® closed coil, American Orthodontics, Sheboygan, WI), according to the sliding technique with a 0.019 × 0.025-inch SS wire. A retraction force was measured every two weeks using an intraoral force gauge (model 040-711-00) from Dentaurum GmbH & Co. KG (Ispringen, Germany). A device containing a battery and an electrical circuit will also output two direct currents (one for each side) with a strength between 5 and 20 μA for each current. The positive pole of the current will be placed on the lateral side of the canine (the area of extraction of the first premolar), while the negative pole will be placed on the medial side of the canine. This circuit is housed within a removable device that the patient must wear for five hours daily.

Removable Electric Maxillary Canine Retraction Accelerator

This electric accelerator was developed by two researchers (M.N.K. and M.Y.H.) in partnership with an electronic engineer (M.A.M.) who designed the electrical circuit. The primary goal was to create a compact, user-friendly device that patients could easily use in their mouths. The appliance applies a low-amperage direct current with controlled parameters to the palatal gingiva around the upper canines, utilizing the electric current to accelerate bone metabolism and expedite tooth movement. It is designed to be secured to the posterior teeth and palatal tissue using retention clasps, ensuring the electrodes remain in place during use. This configuration allows the electric current to pass across the area of the upper canines (mesially-distally).

Device Fabrication and Insulation Method

**Figure 1:**
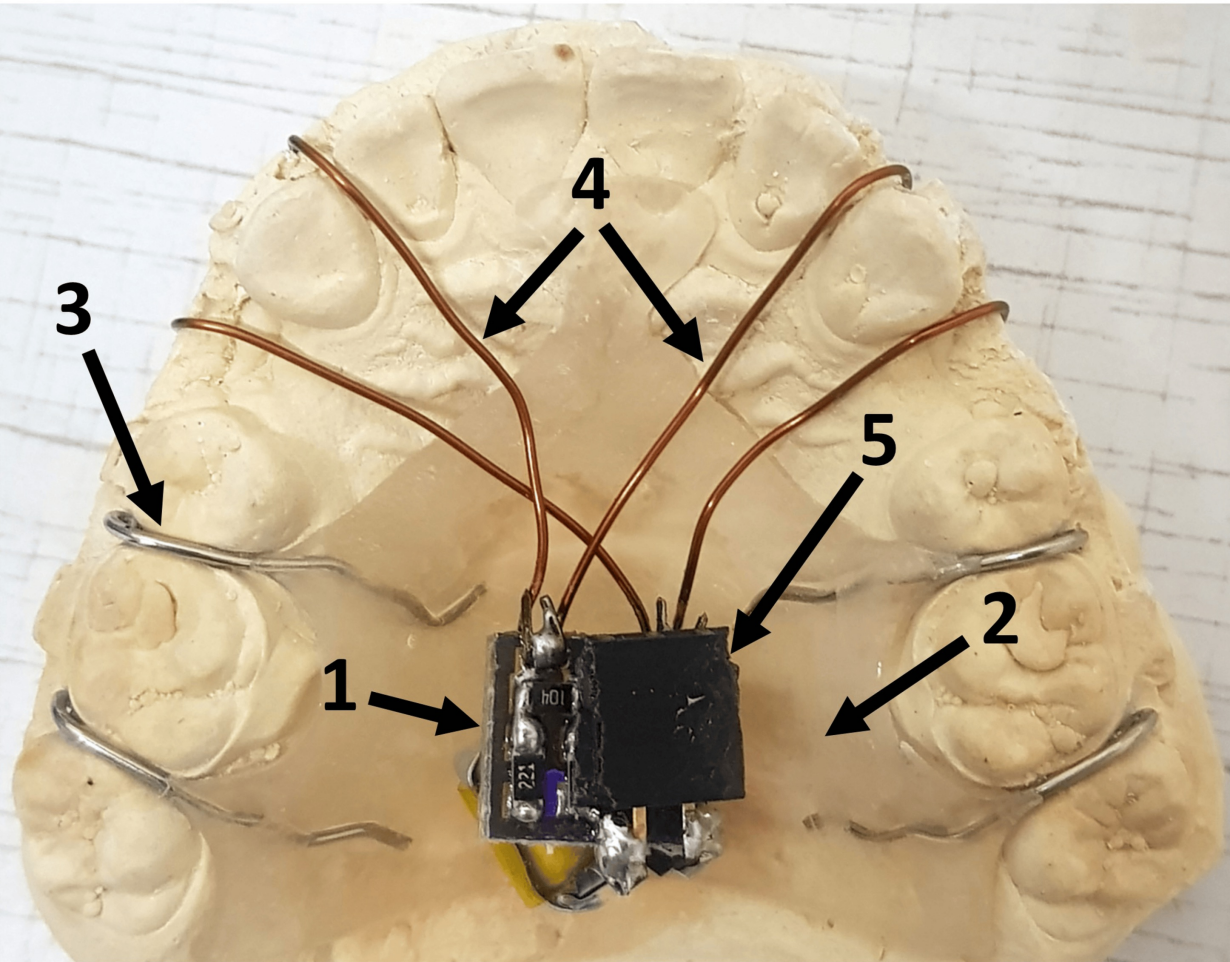
After extending the acrylic layer, the circuit, battery and electrodes are placed in their appropriate places 1: Circuit board, 2: acrylic base plate, 3: retention clasps, 4: electrodes carrying the electric current, 5: charging port

**Figure 2:**
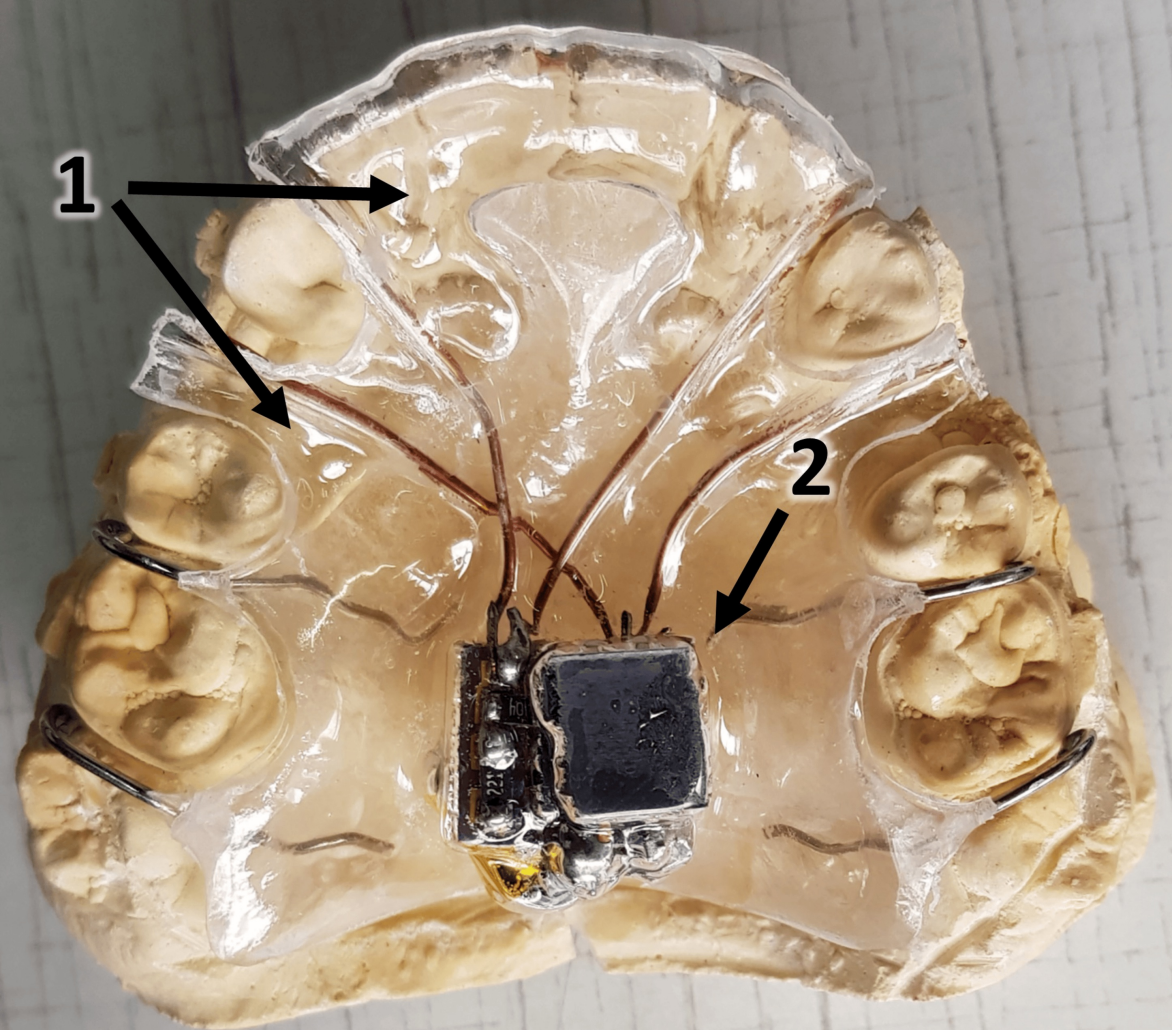
A thin vacuum layer is applied over the device elements to ensure proper fit and insulation 1: The vacuum-formed layer of thermoplastic material above the acrylic base plate, and 2: the insulated circuit and battery

**Figure 3:**
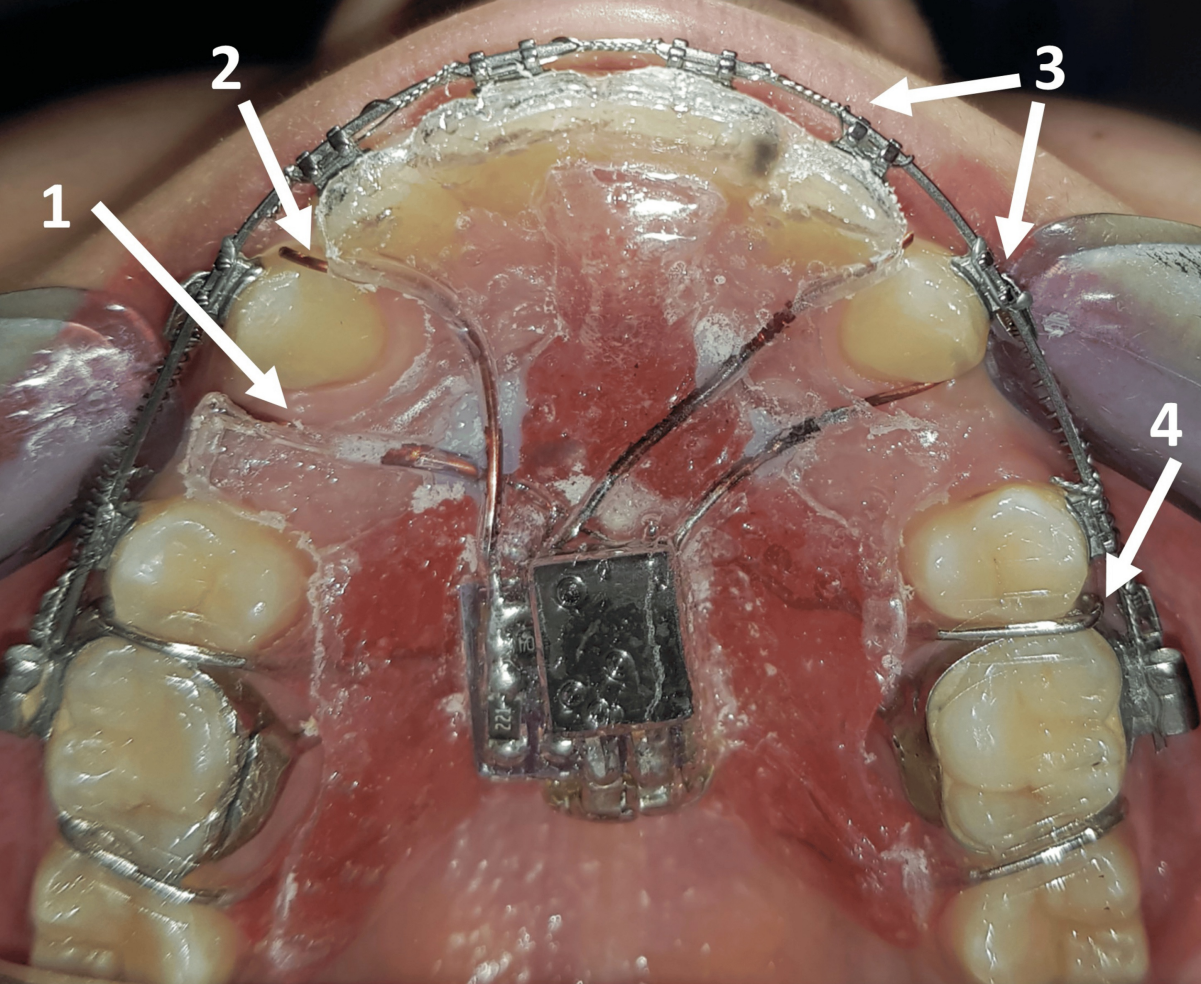
Intraoral application of the device The patient is asked to wear the appliance for five hours daily 1: The trimmed area of the acrylic base plate to provide a space for canine retraction (about 2 mm); 2: the electrodes inserted into their places; 3: the fixed orthodontic appliance (the braces and the archwire); 4: the activated retention clasps to ensure stability

Following the connection of the circuit to the battery, a 1 mm thick acrylic plate is created in the palatal dome area on the patient's plaster model. This plate is centrally positioned, with the positive and negative electrodes placed appropriately: the positive electrode on the lateral side of the canine and the negative electrode on the mesial side. A vacuum-formed plate is then applied over these components. To ensure the canine has a clear path when responding to movement forces, the upper and lateral areas (pathway of the canine) are hollowed out by 2 mm, and this hollowing is periodically adjusted during follow-up sessions. All device components are insulated except for the front ends that deliver the electric current to the mucous tissues. Figures 1-2 illustrate the devices during and after laboratory fabrication, while Figure 3 shows the device in the mouth. Patients are reviewed every two weeks to confirm the device's safety and appropriate current generation.

Primary outcome measure: rate of canine retraction

The movement of the upper canines throughout treatment was assessed using study models. The retraction rate was determined by dividing the distance (in millimeters) by the period (in months). Alginate impressions were used to create model casts at specific intervals: T0, following the completion of the leveling and alignment phase; T1, one month after the initiation of canine retraction; T2, two months later; T3, three months later; and TF, after canine retraction, when a class I canine relationship was achieved. The retraction rate is assessed by measuring the distance between the projection of the incisor tip of the maxillary canine on the maxillary jaw midline and the projection of the medial end of the third palatal fold. This measurement is obtained from digital photographs of the model casts using the ImageJ software, developed by the National Institutes of Health (Bethesda, MD) following the method described by Al-Imam et al. to determine the amount of canine retraction [20]. The monthly retraction rate was determined by dividing the retraction distance by the period.

Secondary outcome measures: safety and patient acceptance

At each appointment, patients were evaluated for any adverse reactions to the electrical stimulation and were asked to report any issues they experienced. At the end of the observation period, patients were asked about their willingness to undergo the treatment again and whether they would recommend this procedure to a friend.

Statistical analysis

Statistical analyses were conducted using SPSS® Statistics software Version 22.0 (IBM® Corp., Armonk, NY). The mean of the movement of both right and left canines at each time point was calculated to determine the average canine movement per month for each patient. Subsequently, the monthly canine movement rate's mean, median, minimum, and maximum values were calculated for all patients at each time point. Finally, the total canine retraction rate was determined by calculating the mean.

## Results

Baseline sample characteristics

After obtaining their consent, seven patients (five females and two males), with a mean age of 20.85 ± 1.34 years, were selected and enrolled in this pilot study. After the follow-up period, the data from these seven patients were analyzed. The patients' pre-treatment characteristics are presented in Table 1.

**Table 1:** Baseline characteristics of the sample at the beginning of the treatment (N=7) SD: standard deviation

Variables	Values
Gender (male/female)	2/5
Mean age (years) ± SD	20.85 ± 1.34
Pre-retraction extraction space, mm	6.23 ± 0.36

Overall retraction rate

The mean canine retraction rate was 1.31 ± 0.3 mm/month during the first month of observation (Table 2). This rate remained nearly constant in the second month at 1.32 ± 0.2 mm/month and slightly decreased to 1.23 ± 0.17 mm/month in the third month. Overall, the mean retraction rate throughout the treatment period was 1.25 ± 0.13 mm/month.

**Table 2:** Descriptive statistics of the upper canine retraction rate (mm/month) T0: at the beginning of treatment; T1: after one month; T2: after two months; T3: after three months; TF: final assessment time SD: standard deviation

Intervals	Mean movement rate ± SD	Median	Max	Min
T0-T1 (1stmonth)	1.31 ± 0.3 mm	1.47	1.6	0.82
T1-T2 (2ndmonth)	1.32 ± 0.2 mm	1.28	1.6	1.19
T2-T3 (3rdmonth)	1.23 ± 0.17 mm	1.2	1.49	1.02
T0-TF	1.25 ± 0.13 mm/month	1.25	1.41	1.04

The safety of the intervention and patient acceptance

The electrical stimulation had no side effects throughout the follow-up period, including burning sensations, irritation, mucosal ulcers, discomfort, swelling, difficulty, or spontaneous bleeding. Patients did not report any harm during the observation sessions. The study participants mentioned that they would recommend this technique to friends as an efficient and convenient method to expedite the orthodontic procedure; they stated that they would have no issues repeating the treatment. Five out of seven patients reported adapting to the canine retraction accelerator was easy, while the remaining two found it moderately challenging.

## Discussion

This preliminary clinical study was designed to assess the efficacy of low-ampere direct current stimulation in shortening orthodontic treatment time. The stimulation was administered to the upper canines using a designed and specifically manufactured electrical device. To mitigate the impact of extraction on the rate of tooth movement by activating the regional acceleratory phenomenon (RAP) and to ensure adequate healing of the extraction sockets, the maxillary first premolars were extracted during the leveling phase, at least two months before canine retraction [21].

This study focused on canine retraction rather than en masse retraction due to several factors. First, a controlled environment is essential for testing the new technique on maxillary canine retraction, as it allows for fine-tuning and monitoring results [22]. Second, canine retraction involves fewer teeth and less complex movements than en masse retraction, simplifying the evaluation of the technique’s effectiveness and safety [22]. Also, starting with a less complex procedure reduces the risk of adverse events or complications, ensuring patient safety [23]. Furthermore, testing the technique on canine retraction first provides baseline data on its effectiveness and safety, which can be used to inform further applications in more complex cases such as en masse retraction [24]. Finally, a stepwise approach permits gradual implementation and refinement of the technique, enabling early identification and resolution of any issues [23]. The trans-palatal arch offered essential support in these moderate cases, ensuring the maintenance of transverse dimensions and aiding in molar derotation when necessary [25]. This approach was crucial for achieving stable and effective orthodontic outcomes [25].

Various methods have been described in the medical literature for orthodontic canine retraction, including sliding and sectional techniques, each with multiple variations [26,27]. This study was conducted using sliding techniques due to their effectiveness in reducing treatment time [26], enhancing aesthetic outcomes [28], and improving patient comfort [29]. This study's protocol for electrical current application drew from Kim et al.'s findings, which showed that low-amperage electrical stimulation of 15 μA for five hours daily could speed up maxillary canine retraction by approximately 30%. However, the design of the fixed device attached to the canine bracket raises concerns regarding its stability and the quality of oral health [17].

This research involves the initial clinical investigation to assess the impact of low-amperage direct current on the maxillary canine retraction rate using a traction device applied only for the necessary duration. Hence, the results were compared with other studies examining different types of tooth movement and various acceleration methods. This study's total maxillary canine retraction rate was 1.25 ± 0.13 mm/month. To assess the efficacy of low-amperage electrical current in accelerating movement, the findings were contrasted with findings from other studies investigating the retraction rate of canines using the sliding technique on 0.019 × 0.025-inch SS wire with conventional brackets and a coil spring applying a force of 150 g without any acceleration method. Tiwari et al. reported a rate of 0.83 ± 0.25 mm/month [30], while Oz et al. observed a rate of 0.95 ± 0.31 mm/month [31] and Husain and Sundari found it to be 0.82 ± 0.39 mm/month [32]. As a result, the movement rate increased by 0.23 to 0.41 mm per month above the normal rate, representing an acceleration of 22% to 33% when low-amperage electrical stimulation was combined with orthodontic forces.

Conversely, the findings of our current study align with those of a similar clinical study, which was published only once and assessed the effect of electrical current on accelerating the en masse retraction of maxillary anterior teeth. In that study, the movement rate was 1.02 ± 0.08 mm/month, indicating a 28% increase in movement speed [33]. A straightforward comparison with recent systematic reviews suggests that the acceleration rate achieved through low-amperage electrical stimulation is comparable to or slightly higher than that obtained from other physical methods of accelerating canine retraction [34,35]. However, it is somewhat lower than the rates achieved through surgical methods of accelerating canine retraction [36-38].

The findings suggest that low-amperage current successfully expedited the retraction of the upper canines. However, additional research is required to validate these findings. Both clinicians and patients reported no significant side effects during treatment, suggesting that the electric device is safe and reliable for intraoral use. Nevertheless, further controlled studies with larger sample sizes are needed to validate these results. Moreover, recent research has highlighted the importance of patient-reported outcome assessments in accelerated orthodontics, which should be considered in forthcoming studies [39].

The study results revealed that all patients indicated they would recommend the treatment to a friend and would undergo the procedure again if necessary. This unanimous approval reflects an elevated level of patient acceptance. This may be attributed to the minimal and tolerable pain and discomfort associated with the procedure, which did not interfere with their daily social activities. Furthermore, while only one patient reported moderate difficulty adapting to the electroaccelerator, the rest found it easy to adapt. These findings align with previous research and confirm the high acceptance of the procedure during canine retraction [40].

Limitations

Even though this is the first study to evaluate low-amperage direct current for accelerating maxillary canine retraction, it has several limitations. Pain, discomfort, and functional impairment related to this technique were not systematically assessed. The absence of a control group and the small sample size were primary limitations. We recommend that future studies consider long-term complications such as bone level changes supporting the canine or negative effects on tooth vitality.

## Conclusions

Our findings indicate that low-amperage direct current electrical stimulation could be an effective technique for accelerating orthodontic movement. Specifically, the research demonstrated that maxillary canine retraction was significantly accelerated by using the electrical stimulation device employed in this investigation, with no adverse effects reported. Patients reacted positively to the device, demonstrating its feasibility and acceptability in orthodontic practice. Overall, the electrical stimulation technique described in this study shows promise for reducing the duration of orthodontic treatment, potentially increasing patient satisfaction and treatment efficiency. This innovative approach could lead to shorter treatment times, making it a viable option for future orthodontic applications, pending further validation through clinical trials and additional studies.
